# Experiences with surgical treatment of ventricle septal defect as a post infarction complication

**DOI:** 10.1186/1749-8090-4-3

**Published:** 2009-01-06

**Authors:** Kasim Oguz Coskun, Sinan Tolga Coskun, Aron Frederik Popov, Jose Hinz, Jan Dieter Schmitto, Kerstin Bockhorst, Kathrin Monika Stich, Reiner Koerfer

**Affiliations:** 1 Department of Thoracic Cardiovascular Surgery, University of Göttingen, Göttingen, Germany; 2 Department of Thoracic and Cardiovascular Surgery, Heart and Diabetes Center, North Rhine Westphalia/University Hospital of Bochum, Bad Oeynhausen, Germany; 3 Department of Anaesthesiology, Emergency and Intensive Care Medicine, University of Göttingen, Göttingen, Germany; 4 Department of Medical Informatics, University of Göttingen, Göttingen, Germany

## Abstract

**Background:**

Complications of acute myocardial infarction (AMI) with mechanical defects are associated with poor prognosis. Surgical intervention is indicated for a majority of these patients. The goal of surgical intervention is to improve the systolic cardiac function and to achieve a hemodynamic stability. In this present study we reviewed the outcome of patients with post infarction ventricular septal defect (PVSD) who underwent cardiac surgery.

**Methods:**

We analysed retrospectively the hospital records of 41 patients, whose ages range from 48 to 81, and underwent a surgical treatment between 1990 and 2005 because of PVSD.

**Results:**

In 22 patients concomitant coronary artery bypass grafting (CAGB) was performed. In 15 patients a residual shunt was found, this required re-op in seven of them. The time interval from infarct to rupture was 8.7 days and from rupture to surgery was 23.1 days. Hospital mortality in PVSD group was 32%. The mortality of urgent repair within 3 days of intractable cardiogenic shock was 100%. The mortality of patients with an anterior VSD and a posterior VSD was 29.6% vs 42.8%, respectively. All patients who underwent the surgical repair later than day 36 survived.

**Conclusion:**

Surgical intervention is indicated for a majority of patients with mechanical complications. Cardiogenic shock remains the most important factor that affects the early results. The surgical repair of PVSD should be performed 4–5 weeks after AMI. To improve surgical outcome and hemodynamics the choice of surgical technique and surgical timing as well as preoperative management should be tailored for each patient individually.

## Background

Post myocardial infarction ventricular septal defect (VSD) is a rare but serious complication, which may result in cardiac wall rupture [1]. Surgical intervention is indicated for a majority of patients. The goal of surgical intervention is to improve the cardiac output and to achieve a hemodynamic stability. Complications of acute myocardial infarction that develop within the first 2 weeks after its onset have been associated with a poor prognosis. Cardiogenic shock as a result of involvement of more than 40% of ventricular mass [2], develops in 10% to 27% of these patients [3]. Relative contraindications for cardiac surgery are EF < 30% at rest myocardium, right ventricular (RV) failure, pulmonary hypertension (PHT), mitral insufficiency 3^rd ^degree (MI) and diffuse coronary artery disease (CAD) without a possibility of revascularisation. A VSD will develop in approximately 1% of survivors [4] and generally occurs one week after the acute event [5].

Anterior septal rupture is caused by anterolateral infarction when 32% of the left ventricle and 10% of the right ventricle were infarcted. Posterior septal rupture is caused by inferoseptal infarction, which results from occlusion of the dominant right coronary artery and occurs in the proximal half of the posterior septum [6] when 21% of the left ventricle and 31% of the right ventricle were infarcted. That is the reason why right ventricular dysfunction as the main determinant [7].

Septal perforation develops on the average 2–3 days after myocardial infarction. Eighty-five percent of patients with acquired VSD or more will die within 2 months without surgical intervention [8]. Significant factors for hospital mortality included: preoperative and evolution of the clinical status, right ventricular function and type of repair. Mortality with only medical treatment is extremely high, over 90% [9]; mortality after surgical repair varies between 19% and 60% in different studies [10,11]. Due to of necrotic myocardium and friable endocardial tissue, the suture of the Dacron patch is difficult with a high risk of recurrence of the VSD and subsequent mortality [12]. The first surgical repair of post infarction VSD was performed by Cooley in 1957 [13]. Daggett and colleagues [14] reported satisfactory results, including reduced mortality and improved cardiac function. In 1990, Komeda and coworkers [15] offered a new concept of surgical repair of post infarction VSD, which they called the "infarct exclusion method".

The purpose of this retrospective investigation concerning post infarction ventricular septal defect (PVSD) was to summarize clinical results by this life-threatening complication in our high-volume cardiac surgery centre North Rhine-Westphalia, Germany.

## Methods

### Study population

Between 1990 and 2005, 41 consecutive patients underwent a surgical treatment because of post infarction ventricular septal defect (PVSD). Preoperative characteristics included gender, age, localisation of the (PVSD), VSD diameter, mean pulmonary artery pressure (PAmean), L-R shunt (L-R), ejection fraction (EF), requirement for intraaortic ballon pump (IABP) and preoperative thrombolysis therapy due to myocardial infarction, (Table 1). Preoperative echocardiographic investigations (inclusive VSD diameter) were performed through the cardiologist at the time of admission, and postoperative echocardiographic investigations were performed three days after the surgery.

**Table 1:** Preoperative characteristics

Characteristics	Patients (n = 41)
Male/Female	30/11
Age (mean y)	68 ± 8
Anterior VSD	27
Posterior VSD	14
VSD Diameter (mm)	27 ± 12
PAmean (mmHg)	32 ± 6
L-R shunt (ml)	58 ± 20%
EF (%)	47 ± 14%
Time Interval AMI-VSD (d)	8.7
Time Interval VSD-OP (d)	23.1
Time Interval AMI-OP (d)	32 ± 25
IABP pre OP	19
Pre OP Intubation	8
Pre OP Thrombolysis	13
Pre OP Cardiac Shock	29

The time intervals from infarct to rupture and from rupture to surgery were analysed. The outcome and the mortality of all patients were collected and are summarized in Table 2. In all patients VSD patch closure was performed using Teflon or bovine pericardium. In all cases the ventricular septal reconstruction included resection of the infarcted area together with the ventricular septal reconstruction. Moreover, in 22 patients concomitant coronary artery bypass grafting (CAGB) was performed. All patients who left hospital were followed up by personal examination or by information received from the referring cardiologist. Data obtained included survival.

**Table 2:** Outcome of the patients

Characteristics	Patients (n = 41)
Residual Shunts (n)	15
Re-OP (n)	7
Concominant CABG-OP (n)	22
All over hospital mortality	32%
30 day mortality	34%
Anterior VSD mortality	29.%
Posterior VSD mortality	42.%
Mortality	
Emergent surgery within 3 days after AMI (n = 5)	100%
Survival	
Surgery after AMI 36 days	100%

### Statistical analysis

Statistical analysis was performed by using commercial statistic software (Statistica 5.1.^®^, StatSoft Inc., Tulsa, OK, USA). Data are expressed as mean ± SD for continuous data and as percentages for categorical data. Patient survival rates were calculated according to the Kaplan-Meier life table method.

## Results

Our patient cohort consisted of 41 patients with PVSD. We found a predominance of male sex compared to female (m = 30/female = 11). The mean age of PVSD patients at follow-op was 68 ± 8.2 years and ages range from 48 to 81. The VSD was anterior (ant) in 27 patients and posterior (post) in 14 patients. Twenty-nine were in cardiogenic shock and the preoperative NYHA classification was III-IV when operated. Preoperative IABP support was used in 19 patients, 8 were ventilated, and 13 patients had thrombolytic therapy. Preoperative mean PA value was 32.09 ± 6.2, L-R shunt was 57.82 ± 20.21%, and EF was 47.24 ± 14.44%. The mean diameter of ventricle septal defect was 27.17 ± 11.9 mm (min.5 mm to max. 45 mm). Average time between acute myocardial infarction (AMI) and VSD development was 8.7 days and from VSD to surgery was 23.1 days. The interval between AMI and surgery was 32 ± 25 days. Seventeen patients had 1 vessel CAD, 11 patients 2 vessel CAD and 13 patients had 3 vessel CAD, respectively. VSD patch closure was performed in all patients (Teflon or bovine pericardium). In 22 cases patients received a concomitant CABG. Eight of 13 patients who had received a preoperative thrombolysis therapy due to myocardial infarction, survived.

In hospital mortality was 32%. The mortality of urgent repair within 3 days in 5 cases of intractable cardiogenic shock was 100%. All patients who underwent the surgical repair later than day 36 survived. In 15 patients the routinely postoperative echocardiographic investigation revealed a residual shunt. Seven of them were rescheduled three weeks later after the first operation to repeat the VSD closure, because the residual shunt was haemodynamically relevant. Four patients required ventricle assist device postoperative, one patient is still alive. The 30 day mortality was 34.1% for the whole series. Twenty-seven patients are still alive. Mortality in post VSD group was 42.8%, and in the group with ant VSD 29.6% (Table 2).

**Figure 1:**
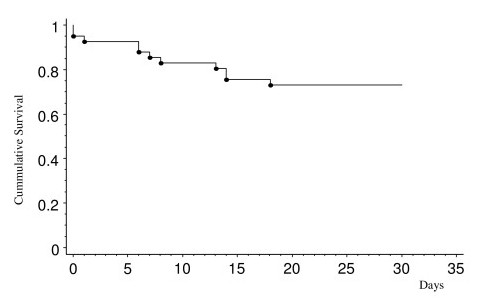
**All over mortality of post infarction ventricular septal defect**.

Overall surgical mortality was significantly higher in the early period after AMI. The cumulative survival analysis is shown as Kaplan-Meier survival function (Figure 1).

## Discussion

PVSD was first described by Latham in 1846 [16] and is known to develop in only 1% of patients with acute myocardial infarction [11]. The most important parameters for prognosis are infarct localization, myocardial function and timing of operation. A prompt diagnosis and immediate cardiac support (IABP or ventricular assist device) is recommended especially in PVSD. Patient survival is directly related to the state of cardiac function at the time of the operation [11].

The development of shock is the most important predictor of survival. Persistence of class IV cardiogenic shock in PVSD is associated with 100% mortality [17]. These findings are in line with our results concerning emergency operation. Worst results were observed in patients with lack of improvement hemodynamic status in spite the use of an IABP. The use of a preoperative balloon pump reflects the condition of the patient before surgery. In patients who are seen more than 2 weeks after onset of AMI, the operative survival is about 70% [18]. The surgical results are improved if the patients can survive for at least 4 weeks, which may be necessary for significant scar formation of the margins of the VSD. One important risk factor is the interval between the myocardial infarction and rupture of the septum. Intervention early is associated with a higher risk [19], explained by the very reduced quality of the tissues near the septal perforation on one hand, and by the reduced functional status of the patients who have to be operated upon very early. Otherwise, Labrousse et al. 2003 [12] recommend an early repair of post infarction VSD, even when the patient is in cardiogenic shock. Moreover, the authors described that the delay of surgery was directly correlated to the preoperative status because a lots of the patients die before surgery. Further, they claimed the ethical problem to delay the operation. However, in their study all late repairs of PVSD were excluded and it could be conceivable, that the mortality rate in "late repairs of PVSD" would achieve a different mortality rate.

Our results concerning the emergent surgery within 3 days after AMI are poor. However, these five cases were operated as ultimo ratio under poor hemodynamic conditions. Comparison of mortality between different institutions remains unreliable. Indeed, patients are usually first referral to cardiologist centres, and a part of the patients (depending of cardiologist habit) might be considered not suitable and then not referred to surgery. So, a part of the discrepancy in operative results between institutions might be attributed to variable recruitment and the in-hospital mortality reflects in part institutional habits. As in our series, the improvement of hospital mortality in the last years is usually found in other studies [12,20]. The decision, to operate a patient who is in cardiogenic shock should be tailored for each patient individually.

Some studies showed no benefit of CABG while others found evidence for concomitant CABG to be advantageous. It has been shown that concomitant myocardial revascularization decreases operative mortality and improves long-term survival [21]. We could not prove any influence of concomitant CABG on late survival of our patients, but patients who have multivessel disease should be routinely revascularised [22].

Cardiogenic shock prior to surgery influenced early survival and preoperative IABP increases cardiac output and decrease the left to right shunt with an improvement coronary perfusion. The need for mechanical ventilation was not a result of delaying operation but to aid in management of pulmonary oedema and/or cardiogenic shock. A longer time between AMI and surgery favoured survival. Time period from AMI to VSD seems to be a significant factor of survival. It is clear that the higher mortality in patients operated on early is also due to the seriousness of hemodynamic conditions which do not allow any delay in surgical treatment. Higher mortality reported in posterior VSD can either be related to greater technical difficulties associated with surgical repair or to a higher incidence of right ventricular failure [19,20]; the size of the left-to-right shunt is inversely correlated with the extent of infarction and directly correlated with residual ventricular function. Patients who survived operative repair had larger shunts on. Survivors had better left ventricular function preoperatively. Chronic VSD is easier to repair since the septum is well scarred and the patch can be securely sutured [23]. Moreover, our results show an increased mortality in patients who had been underwent a thrombolysis preoperative. This result is line with the finding of Crenshaw et al. [11].

In literature, VSD recurrence appeared between 10% and 40% [24] due to perioperative failure of the repair, especially in the posterior localization, because the technical repair there is more challenging. The pathogenesis of residual shunts may be due to incomplete closing of the shunt at operation under certain conditions. Our results are similar concerning VSD recurrence.

Taken together, surgical intervention is indicated for a majority of patients with mechanical complications. Cardiogenic shock remains the most important factor that affects the early results. The surgical repair of PVSD is mandatory. After confirming diagnosis and evaluation of coronary arteries prompt attempts should be done to stabilize the patient since PVSD closure should be performed 4–5 weeks after AMI. LV dysfunction, time interval between VSD-OP and associated organ functions are predictors of operative mortality. Patients who benefit most from the operation were those with a normal postoperative contraction pattern, where ejection fraction improved. These patients present a satisfactory survival and quality of life.

Recently, some authors had reported about a new therapy of a postinfarction VSD with an interventional acute VSD closure [25-27]. However, to date our knowledge postinfarction VSD closure with the less invasive technique still remains limited. Furthermore, there are no data about long term efficacy which could compare the results of surgical closure. This could be a promising therapy and may offer an alternative to surgery.

It is important to note that this study suffered from important limitations. The main limitation of this study is the retrospective nature of our work. Moreover, our study population was smaller than multi-centre studies. However, our single centre observation study has comparatively a relative large number of patients undergoing a PVSD reconstruction between 1990 and 2005. Thus, this study has the strength to show intuitional experiences in patients undergoing a PVSD reconstruction over a long period.

## Conclusion

We recommend a delay of operation, because the mortality in patients who underwent a surgical repair of the PVSD in first 2 weeks after AMI is extremely high. Early repair and posterior rupture are predictors of early mortality; safe operation is feasible about 2 weeks after perforation of the septum. But of course, in patients with cardiogenic shock urgent operation becomes necessary and the choice of surgical technique and surgical timing as well as preoperative management should be tailored for each patient individually.

## Competing interests

The authors declare that they have no competing interests.

## Authors' contributions

KOC and STC had helped with surgical procedures, performed data, graphics, and wrote the paper. AFP, JH, and JDS helped with data interpretation and helped to draft the manuscript. KB and KMS performed analysis and statistics. RK co-wrote the manuscript and added important comments to the paper. All authors read and approved the final manuscript.

## References

[B1] Poulsen SH, Praestholm M, Munk K, Wierup P, Egeblad H, Nielsen-Kudsk JE (2008). Ventricular septal rupture complicating acute myocardial infarction: clinical characteristics and contemporary outcome. Ann Thorac Surg.

[B2] Caulfield JB, Leinbach R, Gold H (1976). The relationship of myocardial infarct size and prognosis. Circulation.

[B3] Bolooki H (1990). Surgical treatment of complications of acute myocardial infarction. JAMA.

[B4] Mullasari AS, Umesan CV, Krishnan U, Srinivasan S, Ravikumar M, Raghuraman H (2001). Transcatheter closure of post-myocardial infarction ventricular septal defect with Amplatzer septal occluder. Catheter Cardiovasc Interv.

[B5] Di Summa M, Actis Dato GM, Centofanti P, Fortunato G, Patanè F, Di Rosa E, Forsennati PG, La Torre M (1997). Ventricular septal rupture after a myocardial infarction: clinical features and long term survival. J Cardiovasc Surg (Torino).

[B6] David TE (1995). Operative management of postinfarction ventricular septal defect. Semin Thorac Cardiovasc Surg.

[B7] Hill JD, Stiles QR (1989). Acute ischemic ventricular septal defect. Circulation.

[B8] Davies RH, Dawkins KD, Skillington PD, Lewington V, Monro JL, Lamb RK, Gray HH, Conway N, Ross JK, Whitaker L (1993). Late functional results after surgical closure of acquired ventricular septal defect. J Thorac Cardiovasc Surg.

[B9] Birnbaum Y, Fishbein MC, Blanche C, Siegel RJ (2002). Ventricular septal rupture after acute myocardial infarction. N Engl J Med.

[B10] Barker TA, Ramnarine IR, Woo EB, Grayson AD, Au J, Fabri BM, Bridgewater B, Grotte GJ (2003). Repair of post-infarct ventricular septal defect with or without coronary artery bypass grafting in the northwest of England: a 5-year multi-institutional experience. Eur J Cardiothorac Surg.

[B11] Crenshaw BS, Granger CB, Birnbaum Y, Pieper KS, Morris DC, Kleiman NS, Vahanian A, Califf RM, Topol EJ (2000). Risk factors, angiographic patterns, and outcomes in patients with ventricular septal defect complicating acute myocardial infarction. GUSTO-I (Global Utilization of Streptokinase and TPA for Occluded Coronary Arteries) Trial Investigators. Circulation.

[B12] Labrousse L, Choukroun E, Chevalier JM, Madonna F, Robertie F, Merlico F, Coste P, Deville C (2002). Surgery for post infarction ventricular septal defect (VSD): risk factors for hospital death and long term results. Eur J Cardiothorac Surg.

[B13] Cooley DA, Belmonte BA, Zeis LB, Schnur S (1957). Surgical repair of ruptured interventricular septum following acute myocardial infarction. Surgery.

[B14] Daggett WM, Guyton RA, Mundth ED, Buckley MJ, McEnany MT, Gold HK, Leinbach RC, Austen WG (1977). Surgery for post-myocardial infarct ventricular septal defect. Ann Surg.

[B15] Komeda M, Fremes SE, David TE (1990). Surgical repair of postinfarction ventricular septal defect. Circulation.

[B16] Latham PM, editor Lectures on subjects connected with clinical medicine, comprising diseases of the heart.

[B17] Bolooki H (1989). Emergency cardiac procedures in patients in cardiogenic shock due to complications of coronary artery disease. Circulation.

[B18] Daggett WM, Buckley MJ, Akins CW, Leinbach RC, Gold HK, Block PC, Austen WG (1982). Improved results of surgical management of postinfarction ventricular septal rupture. Ann Surg.

[B19] Moore CA, Nygaard TW, Kaiser DL, Cooper AA, Gibson RS (1986). Postinfarction ventricular septal rupture: the importance of location of infarction and right ventricular function in determining survival. Circulation.

[B20] Skillington PD, Davies RH, Luff AJ, Williams JD, Dawkins KD, Conway N, Lamb RK, Shore DF, Monro JL, Ross JK (1990). Surgical treatment for infarct-related ventricular septal defects. Improved early results combined with analysis of late functional status. J Thorac Cardiovasc Surg.

[B21] Jeppsson A, Liden H, Johnsson P, Hartford M, Rådegran K (2005). Surgical repair of post infarction ventricular septal defects: a national experience. Eur J Cardiothorac Surg.

[B22] Ramnarine IR, Grayson AD (2005). Simultaneous repair of post-infarct ventricular septal defect and coronary artery bypass grafting. Eur J Cardiothorac Surg.

[B23] Madsen JC, Daggett WM (1998). Repair of postinfarction ventricular septal defects. Semin Thorac Cardiovasc Surg.

[B24] Deja MA, Szostek J, Widenka K, Szafron B, Spyt TJ, Hickey MS, Sosnowski AW (2000). Post infarction ventricular septal defect – can we do better?. Eur J Cardiothorac Surg.

[B25] Holzer R, Balzer D, Amin Z, Ruiz CE, Feinstein J, Bass J, Vance M, Cao QL, Hijazi ZM (2004). Transcatheter closure of postinfarction ventricular septal defects using the new Amplatzer muscular VSD occluder: Results of a U.S. Registry. Catheter Cardiovasc Interv.

[B26] Marinakis A, Vydt T, Dens J, Gewillig M, Van Deyk K, Budts W (2007). Percutaneous transcatheter ventricular septal defect closure in adults with Amplatzer septal occluders. Acta Cardiol.

[B27] Szkutnik M, Bialkowski J, Kusa J, Banaszak P, Baranowski J, Gasior M, Chodor P, Zembala M (2003). Postinfarction ventricular septal defect closure with Amplatzer occluders. Eur J Cardiothorac Surg.

